# *Penicillium citrinum* CFAM 521 Isolated From the Amazon Region: A Novel Source of a Fibrinolytic Enzyme

**DOI:** 10.1155/2024/5306083

**Published:** 2024-10-29

**Authors:** Thayana Cruz de Souza, Marcos Gustavo Araujo Schwarz, Daniela Marinho da Silva, Carolina Rabelo Maia, Cláudia Patrícia Mendes de Araújo, Antônio Alcirley da Silva Balieiro, Luiz Antonio de Oliveira, Wim Maurits Sylvain Degrave, Ormezinda Celeste Cristo Fernandes, Leila Mendonça-Lima

**Affiliations:** ^1^Leônidas and Maria Deane Institute, ILMD/Fiocruz, Rua Teresina, 476, Adrianópolis, Manaus, Amazonas 69057-070, Brazil; ^2^Oswaldo Cruz Institute, Fiocruz, Av. Brasil, 4365, Manguinhos, Rio de Janeiro, State of Rio de Janeiro 21040-360, Brazil; ^3^National Institute for Amazon Research, INPA, Av. André Araújo, 2.936, Petrópolis, Manaus, Amazonas 69080-971, Brazil

**Keywords:** fibrinolytic enzyme, PEG/phosphate, *Penicillium*, thrombolytic activity

## Abstract

Fibrinolytic agents are essential in treating thrombosis, playing a critical role in improving survival rates in cardiovascular diseases. Microbial fibrinolytic proteases have emerged as promising alternatives due to their affordability, specificity, lower toxicity, and reduced side effects. Consequently, the search for microorganisms capable of producing these enzymes has gained significant economic importance in the pharmaceutical industry. This study reports and characterizes a novel fibrinolytic enzyme produced by *Penicillium citrinum* CFAM 521, a strain isolated from the Amazon region. The enzyme was purified using a polyethylene glycol (PEG)–phosphate salt aqueous two-phase system (ATPS). The effects of PEG molecular weight, PEG concentration, and phosphate concentration on the protease partition coefficient (K) were evaluated through a 2^2^ full factorial design. The enzyme exhibited both fibrinolytic and fibrinogenolytic activities. After partitioning in a two-phase system with 10% (w/w) PEG and 15% (w/w) sodium phosphate, the fibrinolytic proteases were predominantly retained in the salt-rich bottom phase (*K* = 0.33). The enzyme has a molecular weight of 34 kDa, with optimal pH and temperature at 9°C and 37°C, respectively. Inhibitory analysis confirmed that it is a serine protease, and its activity was enhanced by the addition of Mn^2+^. Notably, the enzyme exhibited no hemolytic activity. Therefore, *P. citrinum* CFAM 521 represents a novel source of fibrinolytic enzymes, highlighting its potential as an alternative for the development of thrombolytic agents.

## 1. Introduction

The production of new drugs from natural sources has been a key incentive for the development of various sectors in the biopharmaceutical industry. Brazil, with one of the world's largest biodiversities, has a great potential to identify molecules that can be transformed into valuable and innovative products [1, 2]. Considering the remarkable potential of the Amazon to harbor a unique and underexplored microbiological wealth research has been conducted to investigate its microbiome in relation to fibrinolytic enzyme production. Fibrinolytic agents play a crucial role as biotherapeutics in the treatment of thrombosis, a leading cause of cardiovascular disease (CVD) [3, 4]. In 2019, CVDs represented 32% of global deaths, totaling 17.9 million, according to the World Health Organization (WHO) [5]. This underscores that CVD was the leading cause of death worldwide in that year.

Fibrinolytic proteases were initially discovered in *Streptococcus hemolyticus* and *Staphylococcus aureus*, bacteria that produce streptokinase (SK) and staphylokinase, respectively [6]. These enzymes, available in various variants, exhibit different degrees of effectiveness and side effects, including immunogenicity [7]. SK is a medication exclusively used in a hospital setting and is the leading fibrinolytic agent in the treatment of thromboembolic diseases, representing a more accessible vital therapy, especially in less affluent healthcare systems globally [8, 9]. However, its production by *Streptococcus β*-hemolyticus imparts antigenic properties, susceptible to causing allergic reactions [10]. Staphylokinases provide a good response to clot degradation, but their short half-life in plasma and production of high levels of antistaphylokinase antibodies such as IgG require efforts to overcome this problem [11].

The most active and available pharmacological forms on the market consist of fibrinolytic agents that activate plasminogen, such as tissue plasminogen activator (tPA), urokinase-type plasminogen activator (uPA), and recombinant tissue plasminogen activator (rt-PA) alteplase. This group regulates the generation of plasmin, produced from plasminogen, responsible for the degradation of fibrin from blood clots [12]. Urokinase, produced from human kidney cell culture, has been used in patients allergic to SK [13]. Alteplase (rt-PA), marketed as Actilyse, is indicated for the thrombolytic treatment of adult patients with acute myocardial infarction and acute ischemic stroke. Despite proven effectiveness, the time factor is limiting, as the drug is effective if administered within the first 3 hours of symptom onset [14]. Additionally, there are various issues with these substances, ranging from serious side effects, such as internal bleeding, allergic reactions, and bradycardia, to high production costs [12].

The development of new research to select organisms that produce these enzymes becomes essential in the pharmaceutical industry in economic terms. In this context, numerous studies have highlighted the ability of different organisms to synthesize fibrinolytic enzymes, including plants, algae, earthworms, snakes, and microorganisms [15–20]. In particular, the latter group has been extensively researched in recent decades, resulting in the identification of potential producers of thrombolytic biomolecules. Regarding bacteria, new strains have been described as alternative sources of fibrinolytic enzymes, such as *Pseudomonas baetica* SUHU25 [21], *Streptomyces radiopugnans* [22], and *Serratia marcescens* CBAM 519 [23]. Bacteria that belonging to the genus *Bacillus* are the most important source of fibrinolytic enzymes, mainly isolated from fermented foods, such as *Bacillus subtilis natto* obtained from Natto, a traditional Japanese soy food [24], and *Bacillus amyloliquefaciens* DC-4 obtained from the Chinese soybean called Douche, synthesizing the enzymes nattokinase and subtilisin DJ-4, respectively [7, 25].

Filamentous fungi, such as *Penicillium chrysogenum* H9 [26], *Aspergillus ochraceus* 513 [27], *Fusarium* sp. PCCC 480097 [28], *Rhizopus chinensis* [29], *Mucor subtilissimus* UCP 1262 [30], *Paecilomyces tenuipes* [31], and *Aspergillus oryzae* KSK-3 [32], also represent a promising source of these enzymes due to their high production and easy recovery from cellular exudates [33]. Although plants and animals produce lower levels of these enzymes, the growing interest in microbial proteases is eloquent due to their short doubling time and the ease of cultivation and genetic manipulation [26]. Given the great potential and increasing applicability of microbial enzymes in drug production, this study reports the characterization of a new fibrinolytic enzyme produced by a strain of *Penicillium citrinum* CFAM 521 isolated from the Amazon region.

## 2. Materials and Methods

### 2.1. Culture Conditions

*Penicillium citrinum* CFAM 521 was obtained from the Amazonian Fungal Collection (CFAM/FIOCRUZ-Brazil). This strain was originally isolated from Amazon soil and has been archived in the collection since 2002. Subsequently, the culture was subcultured on Malt Extract Agar (MEA) in Petri dishes (Ø = 90 × 15 mm) and incubated at 28°C for 7 days.

### 2.2. Molecular Identification

The stock culture was used for DNA extraction. Total genomic DNA was extracted from *Penicillium citrinum* CFAM 521 mycelia using the DNeasy Blood & Tissue Kit (Qiagen) according to the manufacturer's instructions. The internal transcribed spacer (ITS) region, *β-tubulin* (TUB), and the *calmodulin* (CMD) genes were amplified by PCR in a 25-*μ*L reaction mixture containing 2.5 µL of 10X PCR buffer, 0.5 µL dNTP Mix (10 mM) (Invitrogen), 0.75 *μ*L of MgCl_2_ (50 mM), 0.5 µL of each primer (10 *μ*M), 0.3 mL platinum Taq DNA polymerase (5 U/*μ*L) (Invitrogen), and 2.0 µL DNA template. The same PCR cycle conditions were used for all tested genes: (1) 94°C for 5 min; (2) 35 cycles of 30 s at 94°C, 1 min at 55°C, and 2 min at 72°C; and (3) a 2-min incubation at 72°C. The ITS region was amplified using primers ITS1 (5′ TCCGTAGGTGAACCTGCGG 3′) and ITS4 (5′ TCCTCCGCTTATTGA TATGC 3′) [34]. The TUB was amplified using primers Bt2a (5′ GGTAACCAAATCGGTGCTGCTTTC 3′) and Bt2b (5′ ACCCTCAGTGTAGTGACCCTTGGC 3′) [35], and CMD using primers CF1 (5′ GCCGACTCTTTGACYGARGAR 3′) and CF4 (5′ TTTYTGCATCATRAGYTGGAC 3′) [36].

The amplification was confirmed by electrophoresis on 1.5% agarose gel (w/v), stained with GelRed (Biotium). Sequencing was performed on both strands using the primers described above. The Big Dye Terminator Cycle Premix Kit was used, following the manufacturer's protocol, and the products were analyzed on an ABI 3130XL DNA automated sequencer (Thermo Fisher Scientific) from the Genomics Platform at the Leônidas and Maria Deane Institute (ILMD-Fiocruz).

Forward and reverse electropherograms were used to build consensus sequences in Geneious software version 6.0.6 [37]. Low-quality sequences were trimmed, followed by assembly and BLASTn (Basic Local Alignment Search Tool) (https://blast.ncbi.nlm.nih.gov) analysis, restricting the search to the database of type sequences.

### 2.3. Submerged Fermentation

*P. citrinum* CFAM 521 was cultured in test tubes containing MEA medium for 7 days at 28°C. After the incubation period, the spores were collected and counted using a Neubauer chamber to achieve a final concentration of 5 × 10^6^ spores/mL [18]. Experiments were carried out in 125-mL Erlenmeyer flasks containing Manachini solution [38] consisting of (w/v): 0.2% KH_2_PO_4_, 0.1% (NH_4_)_2_SO_4_, 0.1% MgSO_4_·7 H_2_O, 0.09% NaH_2_PO_4_·H_2_O, 0.1% yeast extract, and 0.5% gelatin. Submerged fermentation was carried out on an orbital shaker at 150 rpm and 28°C for 96 h. After this period, the contents were recovered by vacuum filtration using Whatman #1 filter paper, followed by a 0.45-*μ*m filter membrane. The supernatant was used to determine fibrinolytic activity.

### 2.4. Proteolytic Activity

Proteolytic activity was measured following the protocol described by Leighton et al. [39]. The assay employed 0.25 mL of azocasein (Sigma) as the substrate, prepared in 0.2 M Tris–HCl buffer (pH 7.5), combined with 0.15 mL of the enzyme extract. The reaction mixture was incubated at 25°C for 1 h. To terminate the reaction, 1.2 mL of 10% (w/v) trichloroacetic acid was added. The samples were then centrifuged at 8000 × *g* for 10 min at 4°C, and 0.8 mL of the supernatant was transferred to tubes containing 1.4 mL of 1 M sodium hydroxide. Absorbance at 440 nm was subsequently measured. One unit of protease activity (U) was defined as the enzyme amount required to produce a 0.1 increase in absorbance over 1 h. All assays were performed in triplicate.

### 2.5. Fibrinolytic Activity Assessment

Fibrinolytic activity of the crude extract was evaluated using an agar plate assay, based on the method of Astrup and Mullertz [40], with minor modifications. In short, fibrin plates with a 3-mm-thick fibrin gel, 9 cm in diameter, were prepared by combining 10 mL of 0.5% fibrinogen with 0.1 mL of thrombin (100 NIH units/mL). The mixture was poured into a Petri dish and allowed to solidify for 30 min at room temperature. A 20-*μ*L aliquot of the crude fibrinolytic enzyme extract was then spotted onto the fibrin plate and incubated at 37°C for 18 h. The formation of a clear zone on the plate, indicating fibrin hydrolysis, was proportional to the fibrinolytic activity. Each experiment was conducted in triplicate.

### 2.6. Fibrinolytic Activity Quantification

Fibrinolytic activity was quantified as per the procedure by Wang et al. [41]. Briefly, 0.4 mL of 0.72% fibrinogen solution was mixed with 0.1 mL of 245 mM phosphate buffer (pH 7) in a test tube and incubated at 37°C for 5 min. Subsequently, 0.1 mL of a 20 U/mL thrombin solution was added and the mixture incubated for an additional 10 min. After adding 0.1 mL of the enzyme solution, the incubation continued at 37°C, with mixing after 20 and 40 min. At 60 min, 0.7 mL of 0.2 M TCA was added to halt the reaction, and the mixture was centrifuged at 15, 000 × *g* for 10 min. The supernatant (1 mL) was collected, and its absorbance at 275 nm was measured. In this assay, one unit of enzyme activity (fibrin degradation unit per liter) corresponds to an increase in absorbance of 0.01 per minute at 275 nm. All experiments were conducted in duplicate.

### 2.7. Partitioning of the Fibrinolytic Protease of *Pencillium citrinum* CFAM 521 by Aqueous Two-Phase System (ATPS)

All polyethylene glycol (PEG)/phosphate systems were prepared at a mass of 3 g, and the final solution was prepared with a mixture of PEG [6000 g/mol^−1^ (50% w/w)] and solutions of monobasic (KH_2_PO_4_) and dibasic (K_2_HPO_4_) phosphate salts (40% w/w) in 15-mL centrifuge tubes. The crude extract was added to the systems to complete 3 g and then homogenized in a vortex for 1 min. The mixture was allowed to stand for 1 h. After phase separation, the volume of each phase was measured and the protein concentration and protease activity were determined. The bottom phase is rich in salt, while the top phase is rich in PEG.

### 2.8. Experimental Design and Statistical Analysis

A 2^2^ factorial design with 4 replicates was carried out at the central point to determine the influences and interactions of the concentration of PEG (cPEG) and phosphate concentrations (cK_2_HPO_4_) on the partition coefficient (K), the purification factor (PF), and the yield (Y) (Table 1). The model used to describe the data had a quadratic term (lof) to improve the fit. The statistical software used was R Version 4.0.2 [42], using RStudio version 1.1.4, with different packages (tidyverse, rsm, FrF2, sjPlot, and hnp). The significance level used was 0.05.

### 2.9. Protein Concentration

Protein concentration was determined by the Bradford method, as previously described [43] using bovine serum albumin (BSA) as a standard. The experiment was carried out in triplicate. Briefly, after adding Bradford reagent, absorbance at 595 nm was recorded and used to calculate the protein concentration using the standard curve generated with BSA.

### 2.10. Determination of the Partition Coefficient, Recovery, and PF

The partition coefficient (K) of the enzyme was defined as the ratio between proteolytic activity in the top (PAt) and bottom (PAb) phases as shown in the following equation:

(1) $$\begin{array}{c} K=\frac{\text{PAt}}{\text{PAb}}. \end{array}$$

The PF was defined as the ratio of the specific activity in the top phase to its initial value in the crude extract (Ace) before partition as shown in the following equation:

(2) $$\begin{array}{c} \text{PF}=\left(\frac{\text{PAt}/\text{Ct}}{\text{Ace}/\text{Cce}}\right), \end{array}$$

where “Ct” and “Cce” are the total protein concentrations, expressed in *μ*g/mL, in the top phase and in the initial crude extract, respectively.

The recovery of activity was determined as the ratio between the total activity in the top phase and the activity in the crude extract, expressed as a percentage equation:

(3) $$\begin{array}{c} Y=\left(\frac{\text{At}\cdot \text{Vt}}{\text{Ace}\cdot \text{Vce}}\right)x100, \end{array}$$

where “Vt” and “Vce” are the volumes of the top phase and crude extract, respectively.

### 2.11. Sodium Dodecyl Sulfate–Polyacrylamide Gel (SDS–PAGE)

The molecular weight of the enzyme was determined by electrophoresis on a 12% SDS–PAGE according to Laemmli [44], with a standard molecular weight marker (Bio-Rad, Catalog 161-0317). Protein bands were detected after Coomassie Brilliant Blue R-250 staining.

### 2.12. Effect of pH on Protease Activity and Stability

To evaluate the effect of pH on protease activity, the reaction was carried out using 1% (w/v) azocasein as the substrate in the following buffers: 0.1 M citrate (pH 4, 5, and 6), 0.1 M Tris–HCl (pH 7 and 8), and 0.1 M sodium carbonate–bicarbonate (pH 9) [23, 45], with incubation for 1 h at 25°C. For pH stability, the enzymatic extract was preincubated in the aforementioned buffers at 25°C for 24 h. Afterward, an aliquot was taken and enzyme activity was measured as described in Section 2.4. All experiments were conducted in triplicate.

### 2.13. Effect of Temperature on Protease Activity and Stability

The optimal temperature for protease activity was determined by incubating the enzyme extract at various temperatures ranging from 25°C to 80°C, followed by an activity assay under the pH conditions previously identified as optimal. Thermal stability was assessed by incubating the enzyme at temperatures between 25°C and 80°C for 1 h, after which proteolytic activity was measured under the optimal pH and temperature conditions. All experiments were performed in triplicate [23, 46].

### 2.14. Effect of Protease Inhibitors

The impact of protease inhibitors was assessed by testing the following compounds: 10.0 mM methylphenylsulfonyl fluoride (PMSF), 10.0 mM 2-mercaptoethanol, 10.0 mM ethylenediaminetetraacetic acid (EDTA), and 1.0 mM pepstatin A. The enzyme was incubated with each inhibitor at 37°C for 60 min, and residual protease activity was measured. The activity of the control sample, without any inhibitors, was considered as 100% [23, 47]. All samples were tested in triplicate.

### 2.15. Effect of Metal Ions on Protease Activity

Protease activity was assessed in the presence of various metal ions, known as either protease inhibitors or activators. The effects of these ionic solutions were tested at concentrations of 1.0 and 10 mM. The enzyme was incubated for 60 min at 37°C with the following ions: zinc (Zn^2+^), magnesium (Mg^2+^), copper (Cu^2+^), iron (Fe^2+^), calcium (Ca^2+^), manganese (Mn^2+^), sodium (Na^+^), and potassium (K^+^). The residual protease activity was expressed as a percentage of the activity observed in the control sample, which lacked metal ions [23, 48]. All experiments were carried out in triplicate.

### 2.16. Fibrinogenolytic Assay

Fibrinogenolytic activity was analyzed by incubating 150 *μ*L of a 1.0% human fibrinogen solution with 50 *μ*L of the purified enzyme at 37°C for various time intervals (1, 5, 10, 15, 30, 60, and 120 min) [23, 48]. The reaction was terminated by adding a denaturing buffer, and the resulting digested products were analyzed via 12% SDS–PAGE, following the method of Laemmli [44].

### 2.17. Hemolysis Assay

A blood agar plate was prepared consisting of a blood agar base and fresh defibrinated sheep blood, for the hemolysis assay. The purified enzyme (20 *μ*L of a 16 *μ*g/*μ*L solution) was deposited separately into the pre-made perforations in the blood agar plate (*Ø* = 5 mm). After 3-day incubation at 37°C, the plate was analyzed for the presence of a translucent halo. The extracts of the enzyme and the purified enzyme with the same protein concentration of 0.05 mg/mL were analyzed on a blood agar plate [23, 49]. This experiment was performed in triplicate.

## 3. Results and Discussion

### 3.1. Molecular Identification

The fungus used in this study originates from the Amazonian soil and was sourced from the Amazonian Fungal Collection (CFAM, Fiocruz, Brazil). Initially, the research group conducted a screening with 150 fungal samples to assess the production of fibrinolytic enzyme, with *P. citrinum* outperforming due to its remarkable enzymatic production. Subsequently, this fungus was chosen for further tests that involved purification and biochemical characterization. After sequencing and searching for similarity using BLASTn, the sequences of the three markers (ITS, TUB, and CMD) presented 100% identity with the type species *Penicillium citrinum*, deposited in the database of the National Center for Biotechnology Information (NCBI) with the respective access codes: NR_121224.1, GU944545.1, and GU944638.1.

### 3.2. Evaluation of Fibrinolytic Activity

Fibrinolytic activity was assayed using the fibrin plate method, which showed that the addition of the culture supernatant from *Penicillium citrinum* CFAM 521 resulted in the formation of a clear zone, indicating fibrin hydrolysis. The measured fibrinolytic activity was 310.20 ± 4.24 U/mL. These results surpass those reported by Nascimento et al. [30], who produced fibrinolytic proteases using *Mucor subtilissimus* UCP 1262 and *Rhizopus arrhizus* var. *arrhizus* UCP 1295 on different agro-industrial substrates, obtaining proteolytic activities of 78 and 58 U/mL, respectively.

The potential of *Penicillium* to produce proteases is already known [47], but its fibrinolytic capacity has been little explored. El Aassar, El-Badry, and Abdel-Fattah [26] analyzed the variation in fibrinolytic protease production in different nitrogen and carbon sources using the strain of *P. chrysogenum* H9. To date, this constitutes the first report on the production of fibrinolytic proteases by *P. citrinum*.

Other studies using fungi as sources of proteases have demonstrated that these microorganisms are promising fibrinolytic enzyme producers that may be alternatives to current pharmaceutical applications. Shirasaka et al. [32] found a fibrinolytic protease produced by *Aspergillus oryzae* KSK-3, which was indicated for oral fibrinolytic therapy and nutraceutical applications. Likewise, Kim et al. [31] demonstrated that *Paecilomyces tenuipes* produces a fibrinolytic enzyme that may have potential applications in the treatment of thrombosis.

### 3.3. Partitioning of the Fibrinolytic Protease of *Pencillium citrinum* CFAM 521 by ATPS

A factorial design (2^2^) plus central point was carried out to optimize the *P. citrinum* CFAM 521 fibrinolytic protease partitioning using an ATPS. Independent variables studied were the influence of PEG (cPEG) and potassium phosphate (cK_2_HPO_4_) concentrations on enzyme partitioning. The dependent variables analyzed were the partitioning coefficient (K), activity yield (Y), and the PF. As shown in Table 2, the interaction between cPEG and cK_2_HPO_4_ was significant in all three models. In the PF model, the quadratic term (lof) was not significant; however, it remained in the model, presenting a higher *R*^2^ value (0.94), resulting in a better data adjustment.

**Figure 1:**
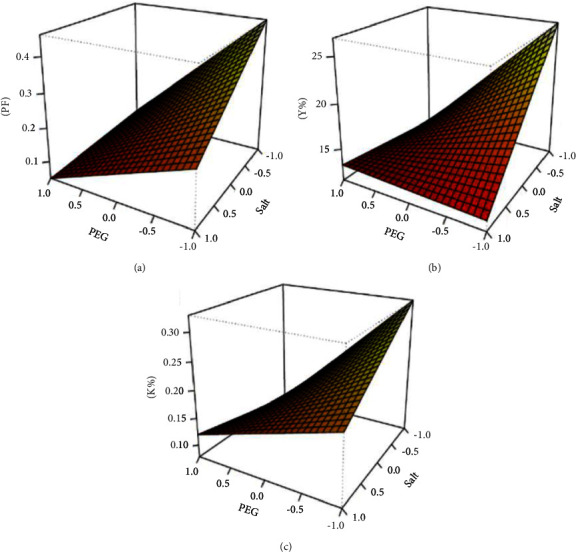
3D graphics of the response variables of the factorial design (2^2^) of *P. citrinum* CFAM 521. Independent variables were the influence of PEG and potassium phosphate concentrations on enzyme partitioning. The dependent variables analyzed were (a) purification factor (PF); (b) recovery (Y); (c) partition coefficient (K). The significance level used was 0.05.

The proposed models indicate a significant negative effect of both independent variables, in which the lowest values of cPEG and cK_2_HPO_4_ gave the highest values in the response variables (Figure 1). Therefore, the significant interaction between cPEG and cK_2_HPO_4_ indicates that the simultaneous decrease of these variables led to an improvement in the PF, yield, and protease partition coefficient. Thus, the system that provided the best results in all three dependent variables was 10% cPEG (w/w) and 15% cK_2_HPO_4_ (w/w). The optimal condition was obtained in Run 1, with PF = 0.41, *Y* = 26.78%, and *K* = 0.33 as shown in Table 3. This result differs from those of the report by Babu, Rastogi, and Raghavaro [50], in which the use of the same PEG/phosphate system showed that the increase in potassium phosphate concentration from 14% to 20% (w/w) resulted in an increase in FP and a decrease in yield (Y).

**Figure 2:**
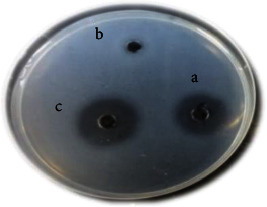
Fibrinolytic activity of the extracts of *Penicillium citrinum* CFAM 521. (a) Crude extract. (b) Partitioned extract—top phase. (c) Partitioned extract—bottom phase.

In all assays, protease activity was preferentially partitioned to the lower, salt-rich phase, with the partition coefficient (K) ranging from 0.07 to 0.33 (Table 3). Therefore, after selecting the best purification conditions (Run 1), the purified extract was again evaluated in a fibrin plate assay to verify fibrinolytic activity in the upper and lower phases compared to the crude extract (Figure 2). It was found that only the lower phase of the purified extract remained with fibrinolytic activity. These results are consistent with those of Sales et al. [51], who evaluated the partition coefficient of fibrinolytic proteases produced by *Bacillus* sp. UFPEDA 485 and found that in all tests, the enzyme preferentially migrated to the lower phase. On the other hand, de Medeiros e Silva et al. [52], using the same purification method for the enzyme produced by *Streptomyces* sp. DPUA 1576, verified at migration to the upper phase, rich in PEG. In general, it is known that the concentration of phosphate in biphasic aqueous systems influences the partitioning by electrostatic charge between biomolecules [53]. In addition, protein partitioning is also influenced by other parameters, such as pH, molecular size, and conformation [54]. Therefore, each molecule is unique and has biochemical characteristics that must be considered during the purification step.

**Figure 3:**
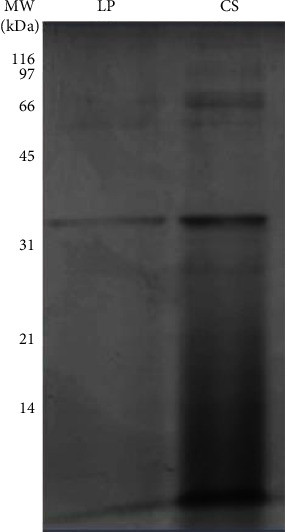
Molecular weight of fibrinolytic enzyme. SDS–PAGE stained with CBB-R250 containing 20 *μ*L of sample from the lower phase (LP) of the aqueous two-phase PEG/phosphate system after partitioning and the culture supernatant (CS).

The fibrinolytic enzyme from *P. citrinum* CFAM 521, recovered from the lower phase after ATPS partitioning, was analyzed by SDS–PAGE. The estimated molecular mass is approximately 34 kDa (Figure 3). Other researchers have reported other fibrinolytic enzymes produced by fungi such as *Pleurotus eryngii* (14 kDa) [55], *Rhizopus chinensis* 12 (18 kDa) [29], *A. oryzae* KSK-3 (30 kDa) [32], *Aspergillus ochraceus* 513 (36 kDa) [27], *Fusarium* sp. CPCC 480097 (28 kDa) [28], and *M. subtilissimus* UCP 1262 (97 kDa) [56]. These data show the structural diversity of fibrinolytic proteases produced by fungi and indicate that research must be continued to discover new activities with different specificities.

### 3.4. Effect of pH on Protease Activity and Stability

**Figure 4:**
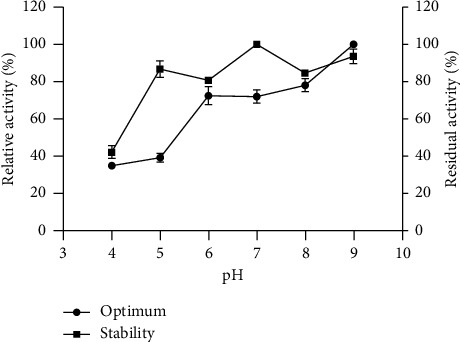
Effect of pH on the proteolytic activity of *P. citrinum* CFAM 521. (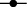) optimal pH: The maximum enzyme activity was set as 100% of the relative activity. (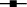) enzyme stability: The effect of pH on enzyme stability was evaluated after 24 h of incubation and expressed as a percentage of the residual activity. Data represent the mean and standard deviation (SD) of three independent experiments (*n* = 3).

The relative fibrinolytic activity (%) was measured across a pH range of 4.0–9.0, with the maximum activity in the curve set as 100%. As illustrated in Figure 4, the optimal pH for fibrinolytic activity was 9.0, while at pH 4.0, the activity decreased by over 70%. To evaluate pH stability, residual fibrinolytic activity (%) was determined, with the unincubated enzyme's proteolytic activity considered 100%. The enzyme displayed significant stability over a wide pH range, retaining more than 80% of its initial activity between pH 5.0 and 9.0 after 24 h of incubation at 25°C.

A similar preference for an alkaline optimal pH has been reported for fibrinolytic enzymes from *Bacillus subtilis* DC27 [57] and *Rhizopus chinensis* 12 [29], which exhibited peak activities at pH 7.0 and 10.5, respectively. In contrast, enzymes from *Mucor subtilissimus* UCP 1262 [56] and *Fusarium* sp. CPCC 480097 [28] showed stability within a pH range of 6.0–9.0. According to Furoni et al. [58], for an enzyme to be suitable for pharmaceutical applications, it must exhibit activity near the physiological pH of blood, which ranges from 7.34 to 7.44. Notably, the enzyme from *P. citrinum* CFAM 521 demonstrated optimal proteolytic activity (approximately 80%) between pH 5.0 and 9.0 (Figure 4), underscoring its potential as a promising candidate for biopharmaceutical applications in human health.

### 3.5. Effect of Temperature on Protease Activity and Stability

**Figure 5:**
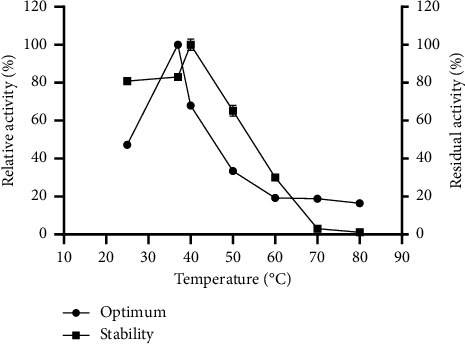
Effect of temperature on proteolytic activity of *P. citrinum* CFAM 521. (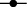) optimal temperature. (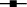) enzyme stability. Data represent average and SD from three independent experiments (*n* = 3).

The relative fibrinolytic activity (%) was evaluated over a temperature range of 25°C–80°C, with the maximum enzyme activity set as 100%. The fibrinolytic enzyme from *P. citrinum* CFAM 521 exhibited its highest activity at 37°C (Figure 5). Residual enzyme activity was measured after 1 h of incubation at various temperatures, with the preincubation activity considered as 100%. The enzyme retained over 60% of its activity after 1 h at temperatures between 25°C and 50°C. Optimal temperatures for proteases from *Mucor subtilissimus* UCP 1262 [30], *Penicillium aurantiogriseum* URM4622 [59], and *Aspergillus oryzae* KSK-3 [32] have been reported to be approximately 40°C, 45°C, and 50°C, respectively.

Regarding thermostability, fibrinolytic enzymes from *Bacillus subtilis* ICTF-1 and *Rhizopus chinensis* 12 [29] were stable within the temperature ranges of 25°C–37°C and 28°C–50°C, respectively, but experienced a loss of activity at higher temperatures. Thus, the enzyme from *P. citrinum* CFAM 521 demonstrated thermal stability at physiological temperatures, highlighting its potential for human therapeutic applications. Additionally, heat-stable enzymes are of considerable interest in research, as thermolabile drugs require stringent conditions for storage and transport to maintain their efficacy and comply with regulatory standards for quality and physicochemical integrity.

### 3.6. Effect of Protease Inhibitors

**Figure 6:**
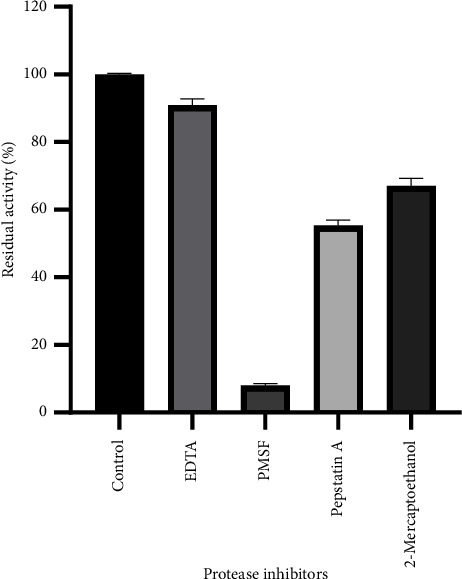
Effect of inhibitors on fibrinolytic activity. Inhibition levels are presented as a percentage of remaining activity relative to the preinhibitor activity (control). Data represent mean ± SD from three independent experiments (*n* = 3).

The enzyme from *P. citrinum* CFAM 521 was significantly inhibited by PMSF, reducing its activity to 8% (Figure 6). Proteases are classified based on the amino acids involved in catalysis at their active sites, such as aspartic, serine, cysteine, or metalloproteases. These results suggest that the fibrinolytic enzyme is a serine protease. PMSF inhibits serine proteases by sulfonating the critical serine residue in the active site, thereby preventing proteolytic activity [60]. Similar findings have been reported for serine proteases involved in fibrinolytic activity from *Mucor subtilissimus* [18], *Bacillus subtilis* (Nattokinase) [57], *Aspergillus oryzae* KSK-3 [32], and *Verticillium* sp. [61]. According to Wu et al. [28], the majority of fibrinolytic enzymes from microbial sources are serine proteases.

Pepstatin A caused a notable inhibition of *P. citrinum* CFAM 521, reducing activity by 45%. This inhibitor specifically targets aspartic residues within the active site of proteases [62]. Similar results were observed by Duarte Neto et al. [59] with the protease produced by *P. aurantiogriseum* URM4622, where enzyme activity was completely inhibited by PMSF and partially inhibited by pepstatin A (55%). These findings strongly suggest the presence of an aspartic residue in the active site of this enzyme, indicating that it is a serine protease with aspartic active site characteristics.

### 3.7. Effect of Metal Ions on Protease Activity

To evaluate the effect of metal ions on proteolytic activity, assays were conducted in the presence of various metal ions. Enzyme activity without any metal salts was used as the control, set at 100%. The addition of Mn^2+^ at 10 mM significantly enhanced proteolytic activity, resulting in a 616% increase (Table 4). In contrast, protease activity decreased in the presence of Cu^2+^ (10 mM). Assays with metal ions at 1 mM did not show any significant changes in enzyme activity. These findings align with the study by Yogesh and Halami [63], which demonstrated that the serine metalloprotease from *Bacillus circulans* also exhibited reduced activity in the presence of Cu^2+^, with residual enzyme activity dropping by more than 50%, while Mn^2+^ positively influenced catalytic activity. Similarly, Mn^2+^ has been shown to enhance the activity of fibrinolytic enzymes from *Serratia marcescens* CBAM 519 [23], *Bacillus cereus* RSA1 [64], and *Aspergillus oryzae* KSK-3 [32]. This enhancement could be attributed to improved substrate binding in the presence of these metal ions or to conformational changes in the enzyme structure due to electrostatic alterations [30].

### 3.8. Fibrinogenolytic Assay

**Figure 7:**
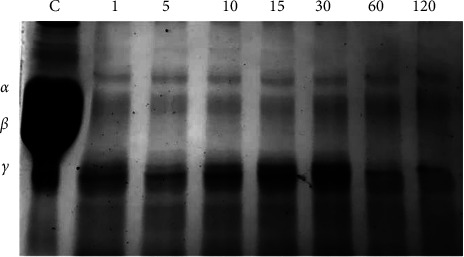
Fibrinogenolytic activity of the enzyme analyzed by SDS–PAGE after the time intervals (in min) indicated above lanes. Line C: control (fibrinogen) with *Aα*, *Bβ*, and *γ* chains indicated to the left.

The fibrinogenolytic assay of the enzyme from *P. citrinum* CFAM 521 showed that the bands *Aα*, *Bβ*, and *γ* disappeared after 1 min (Figure 7) and that all fibrinogen chains (*Aα*, *Bβ*, and *γ*) were susceptible to the cleavage of fibrinolytic enzymes, indicating that it has fibrinogenase-like activity. This hydrolytic pattern was similar to that reported for other purified fibrinolytic proteases from *Fusarium* sp. CPCC 480097 [28], *Codium fragile* [15], and *S. marcescens* CBAM 519 [23]. However, it differed from enzymes found in *B. subtilis* HQS-33 [49] and *B. circulans* [63], in which the *γ* chain exhibited resistance to degradation. The proteolytic degradation of *Aα* chains implies less stability of the clot, while proteolytic degradation of *γ* chains translates into less interaction of fibrinogen with platelets [65]. A decrease in fibrinogen levels contributes to a reduced risk of thrombosis. Consequently, this protease presents itself as a potential candidate for application in thrombolytic therapy and as a preventive measure against the formation of blood clots [23].

### 3.9. Hemolysis Assay

**Figure 8:**
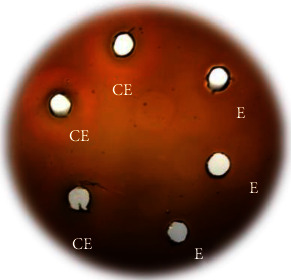
In vitro hemolysis assay on blood agar. (CE) 500 *μ*g of crude extract and (E) 500 *μ*g of enzyme recovered from the bottom phase after ATPS partition were added to slots in blood agar plates and incubated at 37°C for 3 days. *N* = 3.

The effects of the crude extract and the enzyme recovered from the bottom phase after ATPS partitioning were investigated to assess whether the enzyme induced hemolysis. The crude extract exhibited hemolytic activity on blood agar plates, while the enzyme from the lower phase showed no visible hemolytic effect (Figure 8). These findings align with the studies by Huang et al. [49] and De Souza et al. [23], in which fibrinolytic enzymes from *B. subtilis* and *S. marcescens* CBAM 519, respectively, did not induce hemolysis in vitro, suggesting their potential as safe thrombolytic agents.

Fibrinolytic activators such as urokinase, SK, and tPAs have been widely used in the treatment of thrombosis [15]. However, these therapies have been associated with serious complications, including hemorrhage [56]. Due to these risks, there is increasing interest in identifying alternative sources of fibrinolytic enzymes. Thrombolytic therapy, involving the administration of fibrinolytic enzymes, has demonstrated success in dissolving blood clots and maintaining stable blood flow, highlighting its therapeutic potential [10, 66].

## 4. Conclusion

The results of this study highlight the biotechnological potential of *Penicillium citrinum* CFAM 521, a strain deposited in the CFAM, as a producer of thermostable fibrinolytic protease. The data indicate that this enzyme possesses fibrinolytic and fibrinogenolytic properties. Its biochemical characteristics are promising for human applications, as it remains stable under physiological pH and temperature conditions and does not exhibit hemolytic activity. The enzyme, classified as a serine protease with an approximate molecular weight of 34 kDa, was successfully purified using an ATPS, with the fibrinolytic protease being retained in the salt-rich bottom phase. This method proves effective as an initial step for prepurifying the fibrinolytic protease from *P. citrinum* CFAM 521. Given its favorable properties, the enzyme represents a potential novel candidate for developing treatments for thrombosis. However, while these results are encouraging, further in vivo studies using animal models are necessary to evaluate the enzyme's therapeutic potential, bioavailability, and toxicity more comprehensively.

## Figures and Tables

**Table 1:** Levels of the factorial design (2^2^) variables (different concentrations of PEG [w/w] and phosphate salts [w/w]) with four replicates at the central point.

Variable	Levels		
	−1	Zero	+1
cPEG (%)	10%	15%	20%
cK_2_HPO_4_ (%)	15%	18%	21%

**Table 2:** Analysis of variance applied to the regression models used for the predicted quadratic models for *K*, PF, and *Y* in the factorial design (2^2^) of *P. citrinum* CFAM 521.

Factors	*p* value		
	*K*	PF	*Y*
cPEG (%)	< 0.001∗	< 0.001∗	< 0.001∗
cK_2_HPO_4_ (%)	< 0.001∗	< 0.001∗	< 0.001∗
(lof)	< 0.001∗	0.1104	< 0.001∗
cPEG: cK_2_HPO_4_	< 0.001∗	< 0.001∗	< 0.001∗

^∗^Statistically significant values at *p* < 0.05; *R*^2^ = 97% (K); *R*^2^ = 94% (PF); *R*^2^ = 93% (Y).

**Table 3:** Partitioning of protease from *P. citrinum* CFAM 521 using 2^2^ full factorial designs with four replicates at the central point by the aqueous two-phase system.

Run	cPEG (%)	cK_2_HPO_4_ (%)	PAb (U/mL)	PAt (U/mL)	K	PF	Y
1	10	15	207.30	12.70	0.33	0.41	26.78
2	20	15	242.70	80.00	0.07	0.08	10.42
3	10	21	198.00	37.30	0.19	0.23	11.61
4	20	21	240.00	74.00	0.10	0.04	11.61
5 (C)	15	18	200.00	14.70	0.24	0.24	21.45
6 (C)	15	18	210.70	14.70	0.22	0.24	21.45
7 (C)	15	18	171.30	28.70	0.22	0.23	20.52
8 (C)	15	18	184.70	37.30	0.21	0.22	19.58

Abbreviations: cK_2_HPO_4_, concentration of sodium phosphate; cPEG, concentration of PEG; K, partition coefficient of the proteolytic activity; PAb, proteolytic activity in the bottom phase; PAt, proteolytic activity in the top phase; PF, purification factor; Y, recovery activity (%).

**Table 4:** Effect of metal ions on the fibrinolytic activity of *P. citrinum* CFAM 521.

Ion	Residual activity (%)	
	1 mM	10 mM
Control	100.00 ± 6.28	100.00 ± 3.38
Na^+^	82.47 ± 3.86	82.47 ± 0.00
Ca^2+^	74.57 ± 4.34	91.07 ± 2.41
Fe^2+^	72.85 ± 1.93	161.51 ± 3.86
Cu^2+^	74.23 ± 5.79	50.52 ± 1.45
Mg^2+^	79.73 ± 1.93	81.44 ± 1.45
Zn^2+^	83.85 ± 3.38	84.54 ± 0.00
K^+^	79.04 ± 3.86	68.73 ± 4.34
mn^2+^	88.66 ± 0.97	616.49 ± 4.83

⁣^∗^Data represent average ± SD (*n* = 3).

## Data Availability

The data resulted or analyzed during this study are included in this article. Raw data are available from the corresponding author upon request.
